# Verbal probabilities: *Very likely* to be *somewhat* more confusing than numbers

**DOI:** 10.1371/journal.pone.0213522

**Published:** 2019-04-17

**Authors:** Bonnie C. Wintle, Hannah Fraser, Ben C. Wills, Ann E. Nicholson, Fiona Fidler

**Affiliations:** 1 School of BioSciences, University of Melbourne, Melbourne, Australia; 2 School of Historical and Philosophical Studies, University of Melbourne, Melbourne, Australia; 3 The Hastings Center, Garrison, NY, United States of America; 4 Faculty of Information Technology, Monash University, Melbourne, Australia; Technion Israel Institute of Technology, ISRAEL

## Abstract

People interpret verbal expressions of probabilities (e.g. ‘very likely’) in different ways, yet words are commonly preferred to numbers when communicating uncertainty. Simply providing numerical translations alongside reports or text containing verbal probabilities should encourage consistency, but these guidelines are often ignored. In an online experiment with 924 participants, we compared four different formats for presenting verbal probabilities with the numerical guidelines used in the US Intelligence Community Directive (ICD) 203 to see whether any could improve the correspondence between the intended meaning and participants’ interpretation (‘in-context’). This extends previous work in the domain of climate science. The four experimental conditions we tested were: 1. numerical guidelines bracketed in text, e.g. *X is very unlikely (05–20%)*, 2. click to see the full guidelines table in a new window, 3. numerical guidelines appear in a mouse over tool tip, and 4. no guidelines provided (control). Results indicate that correspondence with the ICD 203 standard is substantially improved only when numerical guidelines are bracketed in text. For this condition, average correspondence was 66%, compared with 32% in the control. We also elicited ‘context-free’ numerical judgements from participants for each of the seven verbal probability expressions contained in ICD 203 (i.e., we asked participants what range of numbers they, personally, would assign to those expressions), and constructed ‘evidence-based lexicons’ based on two methods from similar research, ‘membership functions’ and ‘peak values’, that reflect our large sample’s intuitive translations of the terms. Better aligning the intended and assumed meaning of fuzzy words like ‘unlikely’ can reduce communication problems between the reporter and receiver of probabilistic information. In turn, this can improve decision making under uncertainty.

## Introduction

It is well established that verbal descriptors of uncertainty, such as ‘very likely’, are interpreted in different ways by different people (e.g., [1–6]). Verbal probabilities are more ambiguous than numerical ones, and two people can, and often do, have very different numbers in mind when they hear or read words of estimative probability. People also intuitively use different lexicons, or sets of words, to describe their uncertainty [7]. When information about uncertainty is ambiguous, people’s interpretations are particularly sensitive to context, including how memorable or severe a hazard’s consequences are (e.g. probability of infection versus probability of death) (e.g., [1, 3, 4, 8, 9]). Not only can this mismatch create communication problems between the reporter and receiver of probabilistic information [10], it can also compromise predictive accuracy [11, 12] and undermine decision-making.

Despite the literature advocating numerical over verbal probabilities (e.g., [3, 12, 13, 14]), many organisations and professionals that communicate uncertainty, from the Intelligence Community to doctors, prefer to use words to convey their probabilistic forecasts [6, 13, 15, 16]. Words are often seen as a more appropriate vehicle for expressing vagueness and avoiding false precision [17], and to avoid giving an “illusion of rigor” [18]. In an effort to achieve more reliable interpretation, many such organisations have adopted standardised guidelines. That is, they recommend a consistent set of verbal expressions and provide numerical translations (a suggested probability range) to accompany each of those expressions, usually in a separate table. The use of standards also better allows for feedback to be given over time [19], since precise language that can be numerically translated allows verbal forecasts to be validated [20].

Using numerical guidelines for verbal probabilities should encourage consistency between people and in different contexts, but this is not always the case. Even those who are trained to associate particular numbers with particular phrases, such as weather forecasters, are just as variable in converting probability terms to numbers when faced with different contexts [21]. Another issue with providing numerical guidelines to accompany statements or reports (e.g. risk assessments), is that people don't always refer to them. When Budescu and colleagues [22] administered their online survey asking for numerical translations of verbal probabilities, for example, they found that participants clicked on the link to the guidelines table only 1 in 8 times.

Given the limitations of using guidelines tables to convey standards, what are some alternatives for better communicating verbal probabilities? Budescu *et al*. [22] explored whether different ways of presenting verbal probability information encourage interpretations that are more in line with the intended meaning (see also [23]). The authors presented participants with eight statements containing verbal probabilities extracted from IPCC reports. For example, "It is *very likely* that hot extremes, heat waves, and heavy precipitation events will continue to become more frequent." Each statement was in the form of a single sentence containing one of four target terms: Very unlikely, Unlikely, Likely and Very likely (two sentences for each term). Participants were split into two conditions. One group received the statements with a link to an appendix containing the guidelines table, and the other received the statements with the numerical guidelines bracketed in text. The researchers found that people held better-calibrated interpretations of the IPCC report's intended meaning when presented with guidelines in brackets (40% mean consistency compared with 27%). Bracketing numbers in text clearly improved consistency, although these results still indicate surprisingly low agreement with the guidelines. The authors attribute this to a number of factors, including linguistic vagueness, communicator-listener discrepancy, and motivated reasoning, where pre-conceived beliefs, such as political orientation, drive individual judgements. This result could plausibly also be attributed (at least, in part) to the nature of IPCC guidelines, which Budescu *et al*. [23] also note as potentially confusing. Rather than providing mutually exclusive numerical ranges for each expression, the IPCC guidelines contain overlapping categories (Exceptionally unlikely <1%, Very unlikely <10%, Unlikely <33%, More likely than not >50%, Likely >66%, Very likely >90%, Virtually certain >99%). It is unclear if the range for a given expression should span to the adjoining category or to the extremes of the probability scale. E.g., is the range for 'Likely' supposed to mean 67–90%, or 67–100%? The latter is the intended meaning, but this is not intuitive.

The main part of our study conceptually replicates and extends the Budescu *et al*. [22] study. We compare different formats for presenting guidelines adopted by the US Intelligence Community, the ICD 203 analytic standard [24], which *does* contain mutually exclusive numerical categories, to verbal probability statements extracted from intelligence reports, to see which encourages the most consistent interpretation of the verbal probability expressions. Our study mainly differs from Budescu *et al*. [22] by focusing on a different measure of consistency (described below), and testing two other conditions (tool tip and control) for presenting the verbal probability statements. We also explore whether the presentation effect is more pronounced for particular verbal probability expressions.

Besides presenting analytic guidelines in the best possible way, another way to improve consistency between intended and received meaning is to generate standards that are more in line with the most common intuitions about which numbers best align with given verbal expressions. These may be referred to as 'evidence-based lexicons' [25], and reflect the majority interpretation of selected verbal phrases, for example, the average intelligence analyst thinks ‘very likely’ corresponds to 83% probability. Typically, they are generated using ‘membership functions’[26], which show the degree to which a given numerical probability value (e.g. 27%) can be substituted for a given phrase (e.g. ‘very likely’), expressed over a probability interval (0–1, or 0–100%). A function for a given phrase would assign a membership value of 0 to a given number that does not represent the phrase at all, a membership value of 1 (or 100%) to a given number that wholly represents the phrase, and an intermediate membership value to a given number that represents the phrase to some degree [26]. Ho *et al*. [25] used the approach, together with another method, ‘peak value (PV)’, to construct evidence-based lexicons for intelligence analysts, based on the expressions contained in the lexicons of the U.S. Office of the Director of National Intelligence (ICD 203) and the U.K.’s Defence Intelligence. In order to construct similar lexicons for the climate science domain, they also analysed data from Budescu *et al*.’s [22] study on interpreting terms in the IPCC guidelines.

As an extension of our main study, we also compare people’s ‘context free’ interpretations of verbal expressions (provided in the absence of contextual statements from reports or any reference to guidelines) with the ICD 203 guidelines and highlights any areas where there is good or poor overlap. This is a partial replication of Ho *et al*.’s [25] study, but for a larger and qualitatively different sample. Our evidence-based lexicons are constructed using judgements from a large sample of participants from the general public, rather than a small group of Canadian intelligence analysts (validated with another small group of U.K. intelligence analysts). While Ho *et al*.’s sample captures the individuals who mainly produce intelligence reports containing verbal probabilities, our sample captures those who may consume the information in such reports (e.g. policy and decision makers, and in some cases, the general public).

## Materials and methods

### Participants and procedure

A total of 924 participants were recruited from a pool of 4,122 members of the general public who had previously signed up to receive more information about a larger research project (22% response rate). Participants were mainly from Australia (53%), United Kingdom (8%), Canada (5%), New Zealand (5%), United States (3%), Ireland (2%), and India (2%).

Written consent was obtained from participants, and the research project was approved by the Human Research Ethics Committee of The University of Melbourne, with ethics ID number 1646872.5.

**Fig 1:**
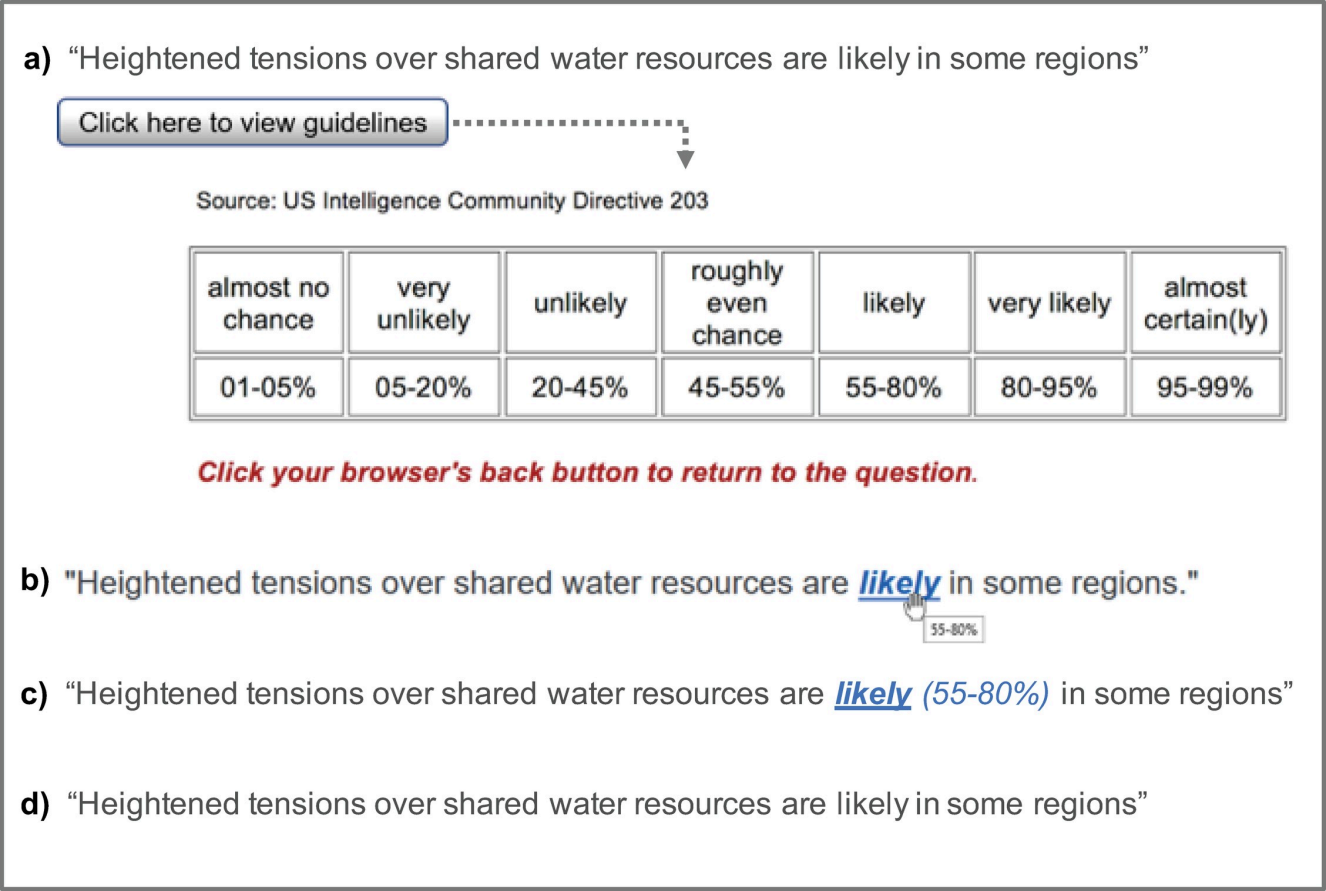
**Four conditions for presenting verbal probability statements: a) click to see a numerical guidelines *table*, b) hover over text to see numerical guidelines in a *tooltip*, c) numerical guidelines presented in text in *brackets*, d) no numerical guidelines provided (*control*).** In all conditions, participants in this example would be asked: “What do you think the authors mean by "likely"?

We measured the consistency of participants’ numerical interpretations of verbal expressions with the numerical translations outlined in the ICD 203 guidelines (shown in Fig 1). Consistency was compared for four different formats (Fig 1) for presenting the relevant verbal expressions from the ICD 203 standard:

- **Table:** The guidelines table, containing a full set of verbal probability phrases and the accompanying numerical probabilities, opens in a separate tab or window when the participant clicks on a clearly marked link to the table.
- **Tooltip:** When the user hovers over the verbal expressions with a mouse, a small image appears, displaying the numerical probability range that accompanies that phrase.
- **Brackets:** Numerical guidelines range presented directly alongside the verbal probability phrase.
- **Control:** Statements contain the verbal expressions only, with no numerical guidelines to refer to.

We first conducted a pilot (*n* = 54) to check for errors and ask people assigned to the tool tip condition if they noticed and used the tool tip function. As only 4 of 12 people (33%) reported noticing the tool tip, we added additional instructions to the main survey for people assigned to that condition to flag the available function.

Participants were randomly assigned to one of the four conditions in an online survey administered through Qualtrics (Provo, Utah, USA). They were first presented with eight randomly ordered statements taken from publicly available post-2015 US intelligence reports that included verbal expressions of probabilities. e.g. “The overall situation in Afghanistan will ***very likely*** continue to deteriorate, even if international support is sustained.” Our four different target verbal probability expressions (very unlikely, unlikely, likely and very likely) were each presented twice. Apart from the format for presenting the accompanying numerical guidelines (or not, in the case of the control), the eight statements were identical for each condition. After reading each statement, participants were asked to provide a minimum, best and maximum estimate of the numerical probabilities that they believe the authors of the report intended to communicate with the verbal probability expression. Next, participants in the Tooltip condition were additionally asked whether they used the tooltip feature (at least once), and if so, approximately how often they used it (out of 8 items). Finally, participants were presented with five items from a numeracy scale [27], five demographic questions, and one political orientation question. Survey materials can be found at https://osf.io/dxng6/. For the Table condition, we also logged IP addresses each time a participant clicked to the guidelines table, using an IP logger tool (IPlogger.org).

Drawing on the same participant pool as the main experimental study, we elicited ‘context-free’ judgements from which we constructed ‘evidence-based lexicons’ for seven verbal probability expressions contained in ICD 203 (as per [25]). *N* = 809 people contributed to this part of the study. Participants were again asked to specify a numeric maximum, best estimate and minimum probability for each of the expressions (i.e., almost no chance, very unlikely, unlikely, roughly even chance, likely, very likely, almost certainly), but in this study, participants were given no intelligence context (i.e., the target terms were not embedded in sentences from intelligence reports). Specifically, they were asked, for example, “To you, what does the probability phrase ‘almost no chance’ mean?”.

### Analysis

For the main experiment, consistency with the guidelines is primarily measured as the percentage overlap between the Lower to Upper (minimum to maximum) ranges specified by participants for a given verbal probability expression (e.g. very unlikely = 1–15%) and the Lower to Upper ranges for that same expression as shown in the ICD 203 guidelines table (where, e.g., very unlikely = 5–20%). Note that in some cases, participants’ responses did not follow the logical ordering of Lower bound < Best Estimate < Upper bound. In these cases, we rearranged participants’ responses into a logical order. We changed the Upper value 137 times, the Lower value 184 times, and the Best estimate 116 times (full code available at https://osf.io/dxng6/). Percentage overlap values between 0 and 100 signify how much the participants’ range corresponds with the ICD 203 range. Negative percentage overlap values signify a complete lack of correspondence and indicate how far apart the two ranges are. The following worked example illustrates our calculations using the example numbers for ‘very unlikely’, given above, i.e., where a participant provides a range of 1–15%, and the guidelines specify a range of 5–20%. Therefore, *Lower*_*1*_ = 1%, *Upper*_*1*_ = 15%; and *Lower*_*2*_ = 5%, *Upper*_*2*_ = 20%, and

Totalrange=max{Upper1,Upper2}–min{Lower1,Lower2}=20−1=19%points (1)

Overlaprange=min{Upper1,Upper2}–max{Lower1,Lower2}=15−5=10%points (2)

Percentageoverlap=Overlap/Total*100=10/19=0.526*100=52.6% (3)

Our dataset has a hierarchical structure, where each participant answered two questions for each verbal probability phrase and they were each assigned to one of four experimental conditions to evaluate how well the percentage overlap for each individual participant is explained by different verbal probability expressions and experimental conditions. Therefore, our primary analysis involves a Bayesian hierarchical model which partitions explained and unexplained variance between participant, phrase and condition and accounts for any non-independence in the data. Our response variable was percentage overlap and we included random intercepts for condition, phrase, and individual participant following the formula:

Percentageoverlap∼1+(1|condition)+(1|phrase)+(1|participantid)

We assigned normal prior distributions to intercepts and regression coefficients (mean = 0, standard deviation = 3), assigned a half-Cauchy prior to the residual variance (location = 0, scale = 5), and assigned a decov prior to all covariance matrices (regularization = 1, concentration = 1, shape = 1, scale = 1). We fitted this model with the rstanarm package [28] in R [29]. We based all inferences on 3 chains of 10000 iterations each.

We conducted an *a priori* power analysis in R (see preregistration), simulating data based on parameters from [22] to determine how likely the hierarchical model was to detect a difference in conditions with our target sample size of 830 participants (which we exceeded). Our power analysis showed that we would detect a true 60% difference in percentage overlap between the different conditions, using 95% credible intervals.

We also calculated statistics that allow for direct comparison with Budescu *et al*. [22, 23]. We plot the data to show means and 95% confidence intervals for an 'inference by eye' comparison [30] of percentage overlap for the four different conditions (verbal phrases collapsed), and also separating out the four different phrases (very unlikely, unlikely, likely, very likely). For further comparison with Budescu *et al*. [22, 23], we calculated the percentage of participants’ best estimates that were within the ICD 203 guidelines and averaged this within each condition. We also re-examined the data in an ‘phrases collapsed’ analysis to exclude participants in the Table and Tooltip conditions who had not used (Table), or reported not using (Tooltip), the functions available to them at least once.

To test whether demographics, numeracy and political orientation influence adherence to the ICD 203 categories, we calculated the correlations and 95% Confidence Intervals (CIs) (for continuous and ranked data) or compared means and 95% CIs (for categorical data) between these variables and participant’s percentage overlap values for the three treatment conditions combined (i.e. excluded the control, as this was the only condition where participants were not given an opportunity to view the ICD 203 guidelines, in any form).

Before analysing the context-free responses from participants (collected after the experimental responses), we conducted a preliminary analysis for possible anchoring effects resulting from providing these judgements *after* seeing a subset of the ICD 203 numerical guidelines in the main experiment. It is possible that participants in three of the experimental conditions (brackets, tooltip, table) might anchor their responses on the guidelines they were given. We therefore calculated the mean and 95% CIs around the best estimates from the context-free responses for each verbal probability expression for each of the conditions separately. As there was no statistically significant difference between the control condition (where participants were not provided with any numerical guidelines to potentially anchor on) and any of the other conditions for any of the four expressions targeted in the main experiment, we concluded that participants in these other conditions did not exhibit significant anchoring.

**Fig 2:**
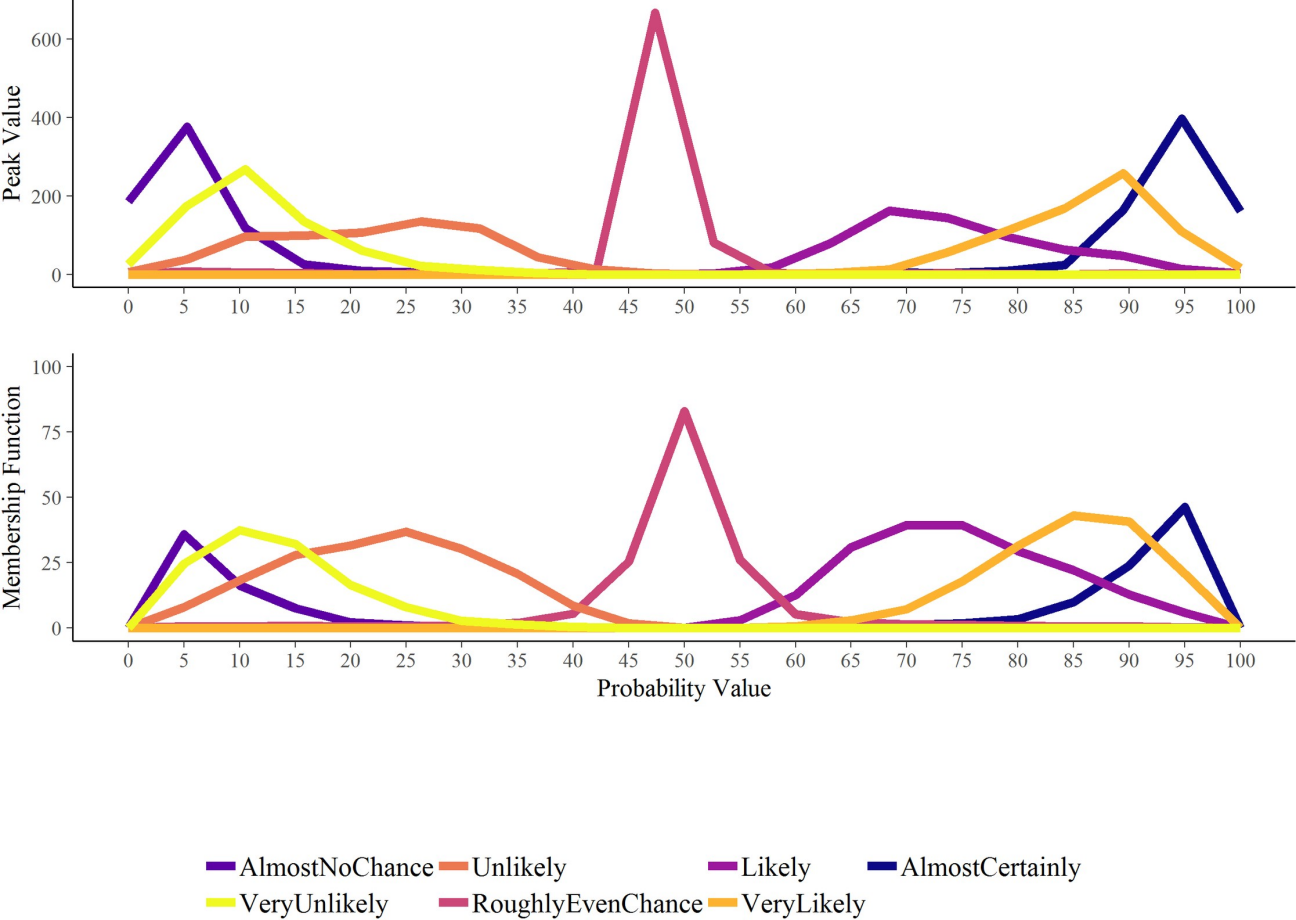
Peak value (PV) and Membership function (MF) methods for charting the frequency (PV) and probability (MF) with which different values are associated with different expressions for our participants.

Following Ho *et al* [25], we used two methods to create evidence-based lexicons from participants’ responses to the context-free questions, these depict the degree to which numerical probabilities can be substituted for each of the verbal probability expressions in ICD 203. The first (‘Peak Value’, PV) charts the frequency with which given best estimate values were assigned by participants to given verbal probability expressions. The cut-off between two adjacent probability expressions (e.g. very unlikely and unlikely) is the point at which the frequency of people associating that number with the two adjacent expressions is the same (where two curves intersect in Fig 2). The Membership function (MF) method we used interpolates between each participant’s minimum, best estimate and maximum for each verbal probability expression to create a triangular membership function representing the extent to which that participant associates all the intervening values with a particular phrase. We then calculate the overall membership function for each phrase by taking the average of the interpolated values for each 5 point increment in probability. As with the PV method, the cut-off between phrases is where a value is equally likely to have referred to each of two adjacent phrases.

## Results

**Fig 3:**
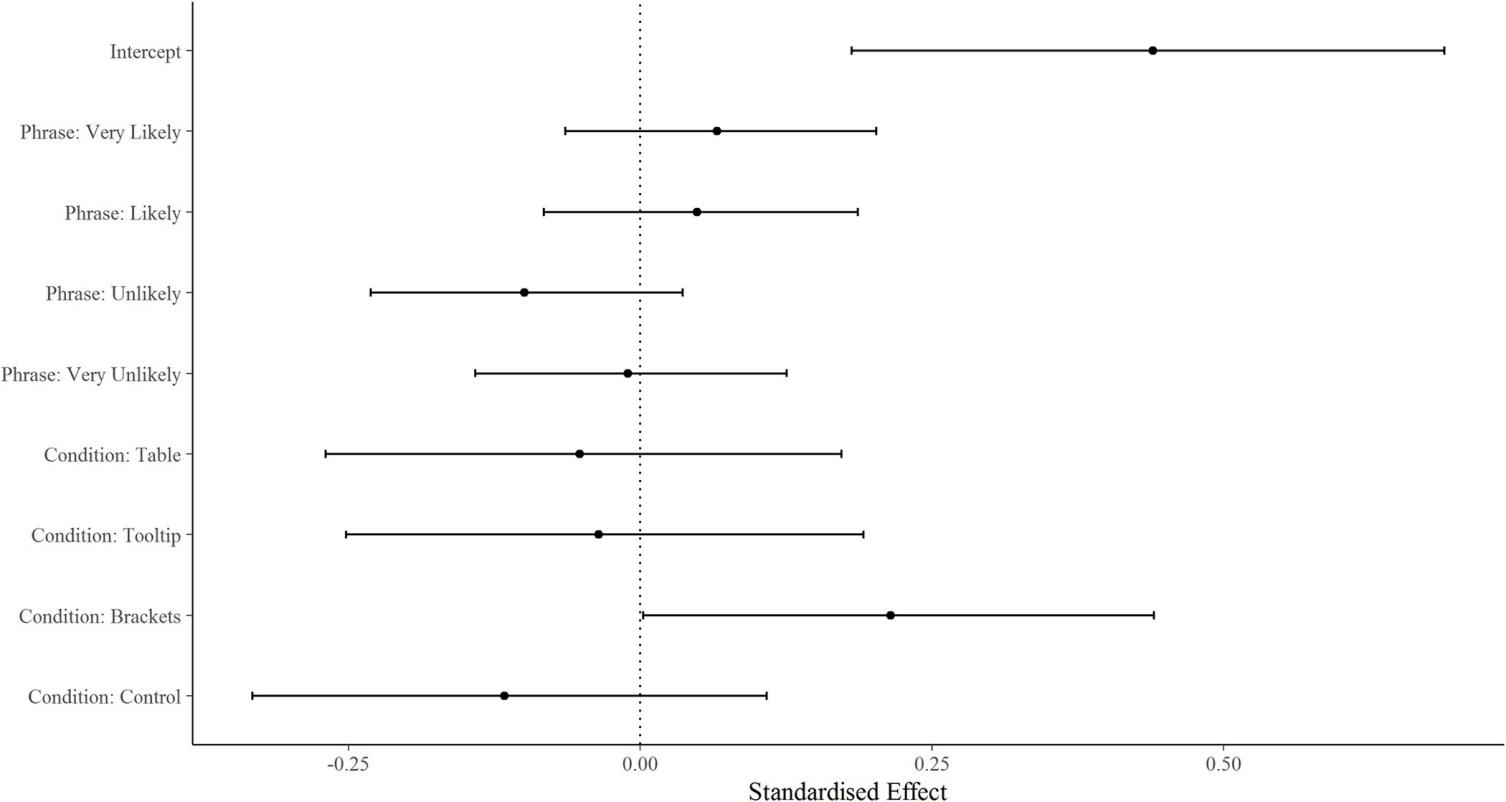
Hierarchical model output showing standardised effects of intercept, condition and verbal probability phrase. Dots represent the mean, bars represent 95% credible intervals. The vertical line at zero reflects the average level of consistency with the ICD 203 guidelines. Condition and phrase judgements above the line perform better than average, and those below the line performs worse than average.

For the main experiment, we included data from all participants who responded to at least one experimental item (Control, *n* = 236; Tooltip, *n* = 225; Table, *n* = 231; Brackets, *n* = 232). Results from the Bayesian hierarchical model (Fig 3) show that all three treatment conditions resulted in participant interpretations of verbal probabilities that were, to varying extents, more consistent with the ICD 203 guidelines than the control, indicated by the improvement in percentage overlap with the guidelines ranges. However, the only condition showing a statistically significant and substantial improvement over the average percentage overlap was the Brackets condition (as the 95% credible intervals do not overlap 0). The model output also shows that, overall, people were least consistent when estimating the phrase Unlikely, followed by Very Unlikely, Likely, and Very Likely.

For comparison with Budescu *et al*. [23] we show that, when percentage overlap scores for all four target phrases were pooled–each participant contributing a single score corresponding to the average percentage overlap across their answers–all treatment conditions showed at least a small statistically significant improvement over the control, indicated by the absence of overlap in any condition’s 95% confidence intervals (in parentheses) and those of the control [30]. Participants in the control group specified ranges with, on average, 32% overlap (29, 35) (95% confidence interval) with the ranges specified in the guidelines. Consistency was highest when numbers were bracketed in text (66% overlap, (62, 70)). There was little improvement over the control for the Tooltip (40% overlap, (36, 43)) and Table conditions (39% overlap (35, 43)).

Only around half the participants in the Table and Tooltip conditions accessed the guidelines available to them. 109 of 230 participants (47%) in the Table condition clicked to the guidelines table at least once, and of those who did, they clicked, on average, for 2 of 8 possible items (questions). Similarly, only 91 of 200 participants (46%) in the Tooltip condition who answered the relevant question reported using the tooltip function at all, and of those, they reported using it on average for 4.5 out of 8 items. When we restricted our analysis to only include ‘active’ participants in those conditions, consistency in the Table condition rose to 44% overlap (38, 51), and in the Tooltip condition, it rose to 49% (43, 55).

**Fig 4:**
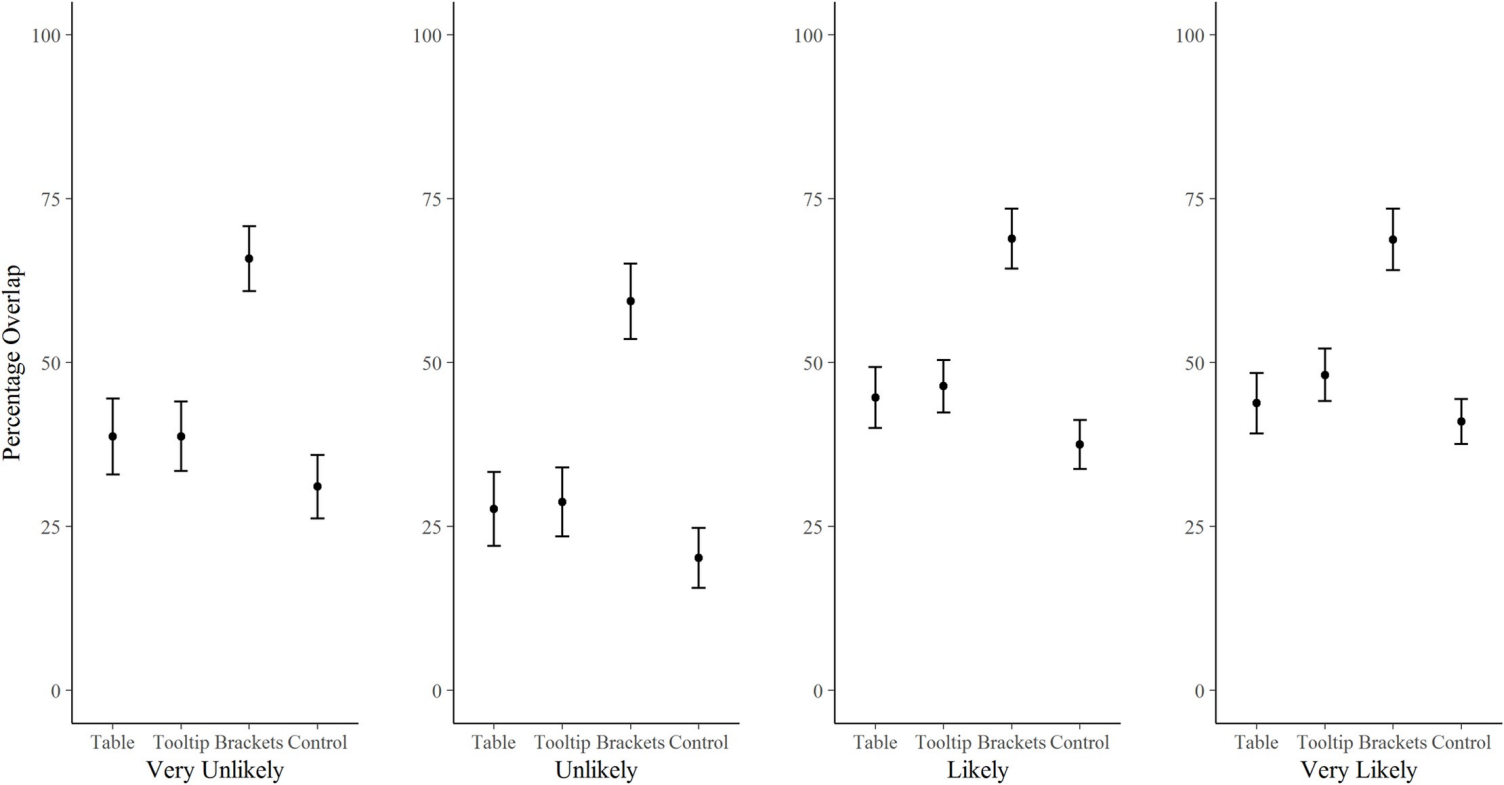
Percentage overlap of participants’ interval judgements in each condition with the respective ranges specified in the ICD 203 guidelines. Results are separated out for each of the four verbal probability phrases.

The main effect of presentation format was stable across each of the four target verbal probability expressions (Very Unlikely, Unlikely, Likely, Very Likely; Fig 4): interval judgements of participants in the Brackets condition overlapped substantially more with the guidelines than those of participants in the other three conditions. The control condition consistently overlapped least with the guidelines, as expected. Overall, correspondence was worst for the ‘Unlikely’ phrase, and the two negatively worded phrases were more variable in terms of percentage overlap of participants’ interval judgements with the guidelines than the positively worded phrases, with standard deviations of: Very Unlikely (44.9), Unlikely (44.5), Likely (36.0), Very Likely (36.4) (see also Fig 4).

**Fig 5:**
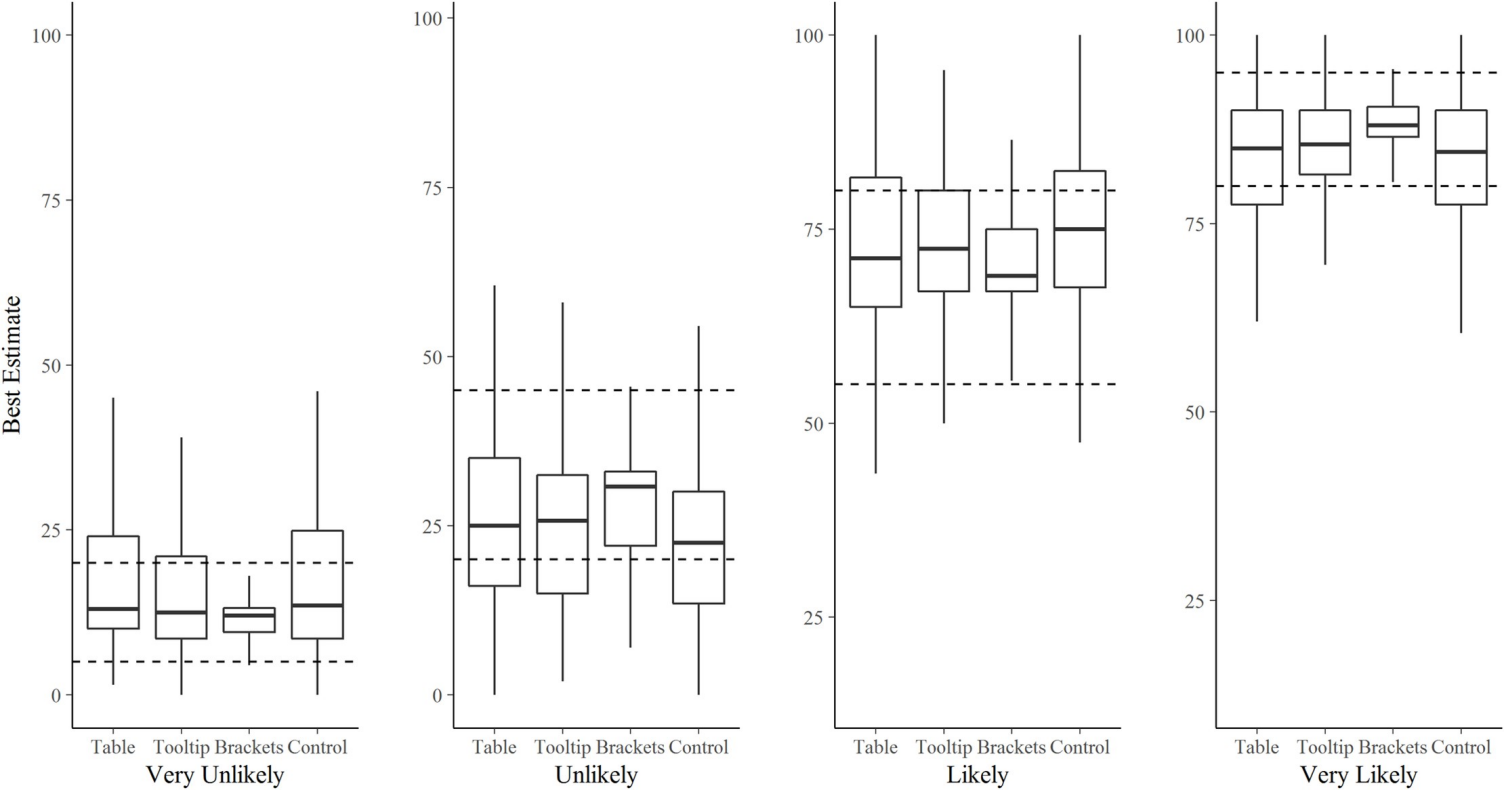
Participants’ translations of each of the four target verbal probability phrases (Very Unlikely, Unlikely, Likely, Very Likely), from words into numerical best estimates. Results are presented by condition. Each boxplot covers ~95% of the distribution. Boxes indicate the central 50% of judgements, the midpoint lines within boxes show the median. Dashed lines represent the ICD 203 guidelines for those target phrases.

The best estimates given by participants in the control condition was within the ICD 203 guidelines 59% (56, 62) of the time, Tooltip 65% (61, 69), Table 64% (60, 68), and Brackets 82% (79, 86). Plotting the spread of best estimates (Fig 5) shows that the variability in interpretation was substantially reduced in Brackets participants, particularly at the extremes of the scale (Very Unlikely and Very Likely). As with the percentage overlap analysis, there was more judgement variation for the two negatively worded expressions, with the standard deviations for participants’ best estimates being: Very Unlikely (21.1), Unlikely (20.7), for Likely (13.0), and for Very Likely (12.9).

We detected a moderate, positive effect of numeracy on percentage overlap with the guidelines (Spearman’s *r* = 0.25 (0.17, 0.33)). We also detected a small, negative effect of age on percentage overlap (Pearson *r* = -0.12 (-0.20, -0.04)), and a negligible positive effect of political orientation (conservatism) on the size of participants’ interval estimates (Pearson *r* = 0.05 (0.02, 0.08). We did not detect a statistically significant effect of gender, education, or country on percentage overlap, nor of political orientation on participants’ best estimates (analysed at both the individual and aggregated question levels). We did not analyse the data on occupation, as responses were provided in free text, and we did not expect to find an effect anyway. In future studies, we would elicit this information in coarse pre-defined categories for ease of analysis.

**Fig 6:**
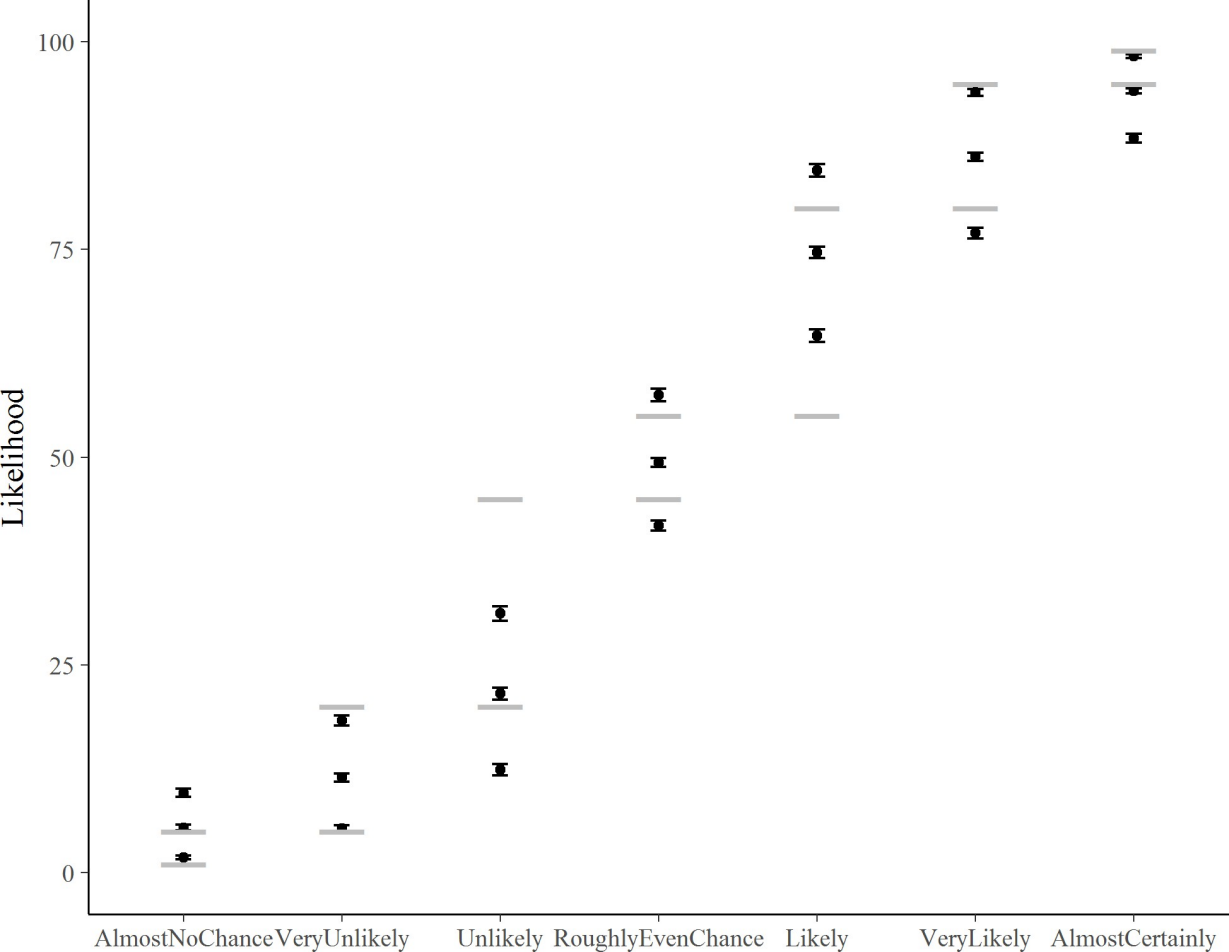
Correspondence between participants’ interpretation of seven context-free verbal probability phrases and the ICD 203 guidelines. The three sets of means (dots) and 95%CIs (error bars) for each phrase reflect the participants’ Minimum, Best estimate and Maximum judgements for that phrase. Dashed lines represent the ICD 203 guidelines.

**Fig 7:**
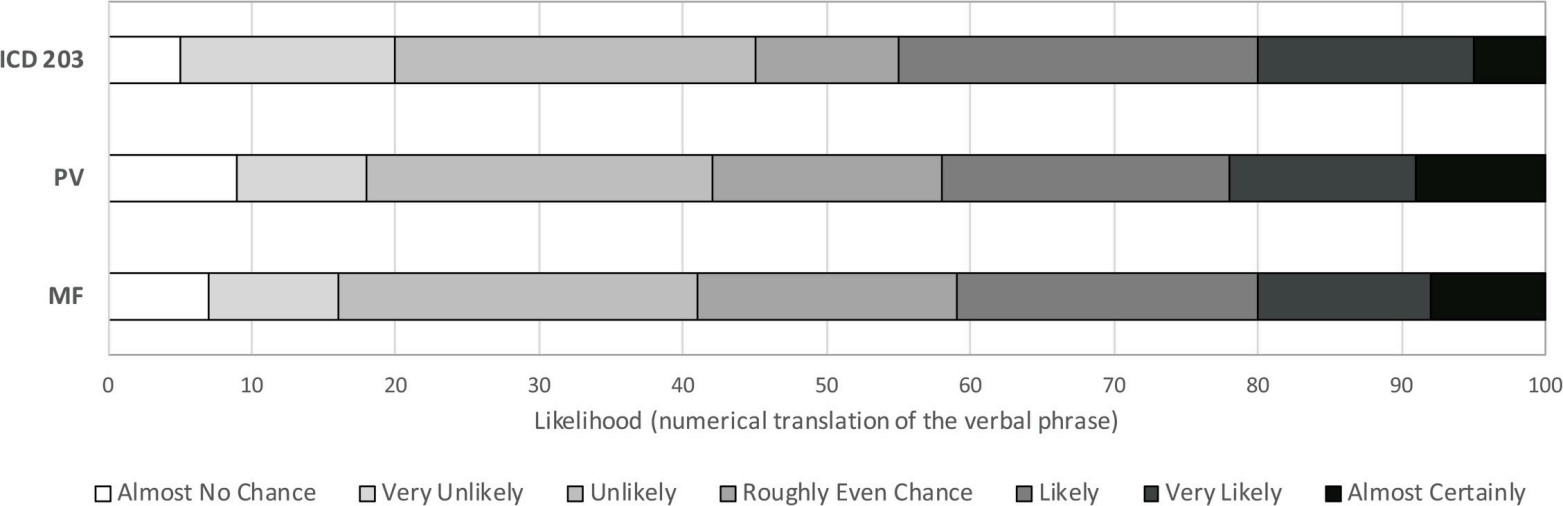
Evidenced-based lexicons obtained using two methods, Peak value (PV) and Membership function (MF), of calculating maximally representative ranges for each of seven commonly used verbal probability phrases, which are also contained in the US Intelligence Community Directive ICD 203 guidelines. For the PV and MF derived lexicons, the cut-off between phrases is where the average participant is equally likely to have assigned that value to each of two adjacent phrases.

Participants’ context-free interpretations of each of seven common verbal probability phrases (also contained within the ICD 203 guidelines) corresponded reasonably well with the ICD203 guidelines (pooled across experimental conditions, Figs 6 and 7). There was almost perfect correspondence between participants’ interpretations and the guidelines for Roughly Even Chance but poorer correspondence for Unlikely and Likely categories. Participants assigned higher probabilities than the guidelines to Almost No Chance, and lower probabilities to Almost Certainly.

Fig 7 shows the ‘evidence-based lexicons’ derived from 924 participants’ context-free estimates for each phrase. Results are similar using both the PV and MF methods for constructing maximally representative numerical ranges for each expression, the former derived from the best estimates, the latter also incorporating the interval estimates provided by participants.

## Discussion

Our results suggest that people do not reliably refer to guidelines unless they are directly in front of them (e.g. translated numerically in text). Just under half of participants in the Table condition accessed a clickable link to the guidelines table, and just under half of participants in the Tooltip condition reported accessing the tooltip guidelines function, despite being instructed to. The improvements in consistency when we restricted our analysis to those active participants suggest that accessing the guidelines through these formats does make a difference. Moreover, the slightly better consistency scores in the Tooltip condition over the Table condition may be explained by active participants accessing the guidelines more frequently in a Tooltip format (4.5 of 8 possible items) than in a click to Table format (2 of 8 possible items).

Our main results are similar to those of Budescu and colleagues [22]. They reported an improvement in mean percentage overlap with prescribed ranges in the IPCC conversion table from 18.5% in their equivalent to our Table condition to 33.6% in their equivalent to our Brackets condition. In a separate study by Budescu *et al*. [23], and using a different measure of consistency (proportion of items per respondent where the best estimate was within the guidelines), the authors reported a similarly sized effect, with mean consistency rates of 21% (Control), 19% (Table), and 30% (Brackets). Our comparable analysis—i.e., the proportion of participants’ best estimates within the guidelines—showed a similar pattern, but with much higher levels of consistency with the guidelines overall, ranging from 59% in the control to 82% in the Brackets condition (and roughly 65% in the Table and Tooltip conditions). Given that we used a more sensitive method for calculating percentage overlap (e.g. where negative rather than zero scores are assigned to judgements that are far outside the guidelines), and the ICD 203 conversion table we tested is less confusing than the IPCC table tested by Budescu and colleagues (containing non-mutually exclusive categories), it is not surprising that our detected effect was larger, and that participants responses were more compatible with the guidelines overall.

Similar to other studies [22, 23, 31], although to a lesser extent, our findings show regression to 50% in the extreme categories, with probabilities at the lower and upper ends of the scale being over- and under-estimated, respectively (in both Part 1 and Part 2). There is clear scope for correcting this regressive pattern at the extremes by bracketing numerical conversions in text, and our results indicate that this is where it may be particularly effective (at least in terms of reducing variability between people, Fig 5). Note, however, that we found the opposite pattern in the least extreme directional categories, Unlikely and Likely, i.e., participants’ judgements of these expressions were systematically *more* extreme than the ICD 203 guidelines table (Figs 5 and 6), even in the Brackets condition (Fig 5). Our participants’ mean best estimates of 29 and 22 for Unlikely and 73 and 75 for Likely (Parts 1 and 2, respectively), were substantially more extreme than those reported in Budescu *et al*. [23]: 44 for Unlikely and 54 for Likely (p. 189). This is despite those categories in the IPCC guidelines table (accessible to two thirds of their participants) being already more extreme (e.g. Likely >66%, with a midpoint of 83%) than the equivalent categories in the ICD203 guidelines table available to our participants (e.g. Likely 55–80%, with midpoint of 62.5%). This difference may also reflect the different ways of presenting the numbers in the two standards, or the different contexts. Harris *et al*. [32] showed that explicitly stating the interval bounds (e.g. 0–33% for Unlikely; 66–100% for Likely) led to less regressive interpretations of those same verbal probability expressions than when the bounds were not explicit (e.g. < 33%; > 66%).

Another interesting pattern we found was that negatively-worded expressions (e.g. Unlikely) prompted judgements that were more variable and less consistent with the guidelines than their positively worded complements (e.g. Likely). This could be seen in the larger standard deviations for participants’ best estimates and lower overall percentage overlap for both the negative phrases compared with both the positive phrases (Fig 4). A similar effect was also uncovered by Smithson *et al*. [33], with negative wording leading to less consensus than positive wording. (As an aside, they also found estimates for negative phrases to be more regressive, but our data does not reflect that pattern). Just as prospects framed in terms of ‘chances of success’ are more attractive than the same prospects framed as ‘chance of failure’ [34], positive phrases reflecting a positive frame (e.g., “it is *entirely possible* that I will succeed”), are perceived to be more optimistic, more correct, and less surprising when the positive outcome actually occurs, compared to if a negative probabilistically equivalent phrase is used (e.g., “it is *not quite certain* that I will succeed”) [35]. Mandel [8] similarly found that verbal probabilities were better discriminated when used for success rather than failure outcomes. Positive phrases prompt the respondent to explicitly focus on reasons for occurrence of an outcome, whereas negative phrases implicitly draw attention to non-occurrence of an outcome, “without actually naming it” ([8, 35], p.70). This may reduce people’s uncertainty, and thus variability, in assigning numbers to positive phrases. Another possible explanation for this effect is that people, as informal forecasters, are not given the opportunity to calibrate unlikely events in the same way as likely events. We tend to register events that happen, more so than those that *don’t* happen. Whatever the reasons for this directionality bias, it affects forecasting, and the decisions that rest on those forecasts [35–37]. For example, a medical treatment framed as having *some possibility* of being effective was endorsed by almost three times the number of participants than when effectiveness was framed as *quite uncertain*, despite these phrases being assigned the same numeric probabilities by a control group [36].

In their studies drawing on IPCC reports, Budescu *et al*. [22, 23] found phrase interpretation to be correlated with ideology, and experience with and beliefs in climate change. We did not elicit an analogue in intelligence and geopolitics (for example, we did not ask respondents about their support for different approaches to foreign policy). We did, however, ask a simple question about political orientation (eliciting participant self-identification on a scale from very liberal to very conservative), and did not detect a correlation with participants’ best estimates for the analogous in-context questions (those based on statements from intelligence reports). Budescu *et al*. [22] also found a correlation between phrase interpretation and education and numeracy. While we did not find a relationship with education, we did detect a moderate, positive effect of numeracy on consistency with the guidelines, and a small, negative effect of age on consistency. The former finding resonates with research showing that highly numerate individuals are less susceptible to framing effects [38], and so may be more stable in their interpretations of verbal probability phrases.

The literature on verbal probabilities describes some opposition in professionals and organisations to reporting numbers alongside verbal expressions of uncertainty [6, 13, 15, 16, 18]. However, since the revision of intelligence standards outlined in the policy document ICD 203 [24], readers of intelligence reports have at least had access to numerical guidelines tables, just not directly in text. Adding a tooltip function for online reports might make numerical guidelines more accessible without occupying space in text, and so may be more acceptable to organisations such as those within the Intelligence Community. For this reason, we had hoped that the tooltip function in our experiment would have been more convincingly effective than it was. Nonetheless, the small improvement it offered over providing an external link to guidelines tables may warrant further consideration. There is also some evidence that resistance to in-text numerical guidelines can be overcome. In a trial with his Canadian Intelligence unit spanning eight years (2004–2011), Barnes [13] promoted the use of numerical translations (0/10 to 10/10) alongside verbal expressions contained within Intelligence reports, e.g. “It is *very unlikely* (1/10) that either of these countries will make a strategic decision to launch an offensive war in the coming six months”. Barnes reported that members of the division were initially resistant to using numbers, particularly percentages, as this may have given the impression that the numbers were calculated or measured scientifically. But he also reported that people became accustomed to using numbers, that it promoted greater attention to the estimative judgements made by analysts, and it allowed more transparency about uncertainty.

Turning to the additional ‘context-free’ results from our study, we can compare the evidence-based lexicon derived from our large, non-expert sample with the ICD 203 guidelines and with the evidence-based lexicon of Ho *et al*. [25]. Consistent with Ho *et al*.’s findings, and with other literature [39], we see that the MF method for deriving the lexicons produces narrower and more extreme numerical intervals at the ends of the verbal probability scale (remote chance and almost certainly), than does the PV method. In this respect, it is more compatible with the ICD 203 standards. We do acknowledge, however, that our method for deriving the MFs differed from the original method proposed by Wallsten and colleagues [26], which constructs functions from participant responses to questions about how well different numeric probabilities (between 0–100%) are described by a spectrum of probability terms, such as ‘doubtful’ or ‘likely’. Our method was based on Ho *et al*.’s [25] reanalysis of Budescu *et al*.’s [22] IPCC interpretation data.

Although extremes are still moderated in our sample compared with the ICD standard, this effect is less evident (for both MF and PV methods) than it was in Ho *et al*.’s sample. Similar to Ho *et al*.’s results, we found that our participants, on average, interpret the central category of the verbal scale (roughly even chance) more broadly than the standards do. Our evidence-based lexicon differs from that of Ho and colleagues in that it reflects the interpretations of lay consumers of reports (rather than analyst consumers or authors). Given that many consumers of reports (such as policy or decision makers) may not be trained in analytical standards, we believe it is useful to compare the ‘natural’ interpretations of commonly-used verbal probability phrases for both types of report consumers. While Ho *et al*.’s results indicated that “intelligence analysts’ conceptions of probability terms simply do not match those of the organizational lexicons” (p.53), we found greater symmetry and compatibility with the ICD 203 standards, perhaps reflecting the larger sample. Our results are more consistent with those of Mandel [8], who found quite good correspondence between the participants’ perceived meanings and a Canadian intelligence unit’s intended meanings of the probability terms, albeit using a standard with nine rather than seven categories. Nonetheless, we did see some deviations from the ICD 203 standard, particularly in the slightly larger ranges indicated for the most central category and the extremes.

It is tempting to argue that standards should be based on how a large number of potential consumers of verbal probability information interpret these terms, rather than definitions posed by a small number of institutional experts in-house. It makes sense that communication of risks and uncertainty improves if standards are developed with the target population in mind (e.g., patients, colleagues, or superiors) [25]. However, that assumes that those consumers are genuinely in touch with how they use those terms themselves. Also, judgements averaged over multiple people are consistently regressive for terms at the ends of the probability scale, suggesting that some words may need to be reserved for the most extreme, precise numeric probability intervals (e.g. 0–5%, or even 0–1%). This is particularly important for institutions and disciplines that routinely deal with predicting rare or almost certain events. A more granular scale in standards such as ICD 203 may give scope for more narrowly interpreted extremes, but only if the consumer can recognize the verbal term as representing the endpoint of a relatively granular scale. There is also a risk that more than seven verbal categories may become too cognitively demanding to distinguish or recall [40, 41], leading to even more variability in interpretation. However, there is evidence that forecasters of intelligence-like events are capable of accurately discriminating nine [42] to twelve [43] numerical categories on a probability scale, even though people only tend to produce five or six verbal terms for probabilities when asked [44].

## Conclusions

The experimental results of our study uncontroversially demonstrated that consumers of words of estimative probability in intelligence reports show high variability when translating these words into numbers. This variability can be substantially reduced by using in-text numerical guidelines. It is difficult to encourage attention to numerical translation guidelines when they are presented in other ways (specifically, in mouse-over tool tips or in accompanying guidelines tables, even if they are easy to access). Our study adds to the evidence that variability and potential biases in interpreting verbal probabilities can only effectively be reduced if numerical translations are highly visible alongside verbal expressions in reports. This would produce less ambiguous forecasts that can be interpreted more consistently, leading to more informed decision making.

The ‘context-free’ responses in our study were used to build an evidence-based lexicon that can be compared with that of Ho *et al*. [25], constructed from and for intelligence analysts. It differs by drawing on a much larger, non-expert sample, to reflect the interpretations of potential non-analyst consumers (rather than analyst generators) of reports. It is useful to see how the differing sample characteristics are reflected in the lexicon. Consistent with previous research, the ends of the probability scale are more moderately interpreted than many institutional guidelines indicate they should be, which is a consideration for disciplines that routinely assess extreme risks using verbal probabilities.
